# Pulsed DC Electric Field–Induced Differentiation of Cortical Neural Precursor Cells

**DOI:** 10.1371/journal.pone.0158133

**Published:** 2016-06-28

**Authors:** Hui-Fang Chang, Ying-Shan Lee, Tang K. Tang, Ji-Yen Cheng

**Affiliations:** 1 Research Center for Applied Sciences, Academia Sinica, Taipei, Taiwan; 2 Institute of Biomedical Sciences, Academia Sinica, Taipei, Taiwan; 3 Institute of Biophotonics, National Yang-Ming University, Taipei, Taiwan; 4 Biophotonics and Molecular Imaging Research Center (BMIRC), National Yang-Ming University, Taipei, Taiwan; 5 Department of Mechanical and Mechantronic Engineering, National Taiwan Ocean University, Keelung, Taiwan; Temple University School of Medicine, UNITED STATES

## Abstract

We report the differentiation of neural stem and progenitor cells solely induced by direct current (DC) pulses stimulation. Neural stem and progenitor cells in the adult mammalian brain are promising candidates for the development of therapeutic neuroregeneration strategies. The differentiation of neural stem and progenitor cells depends on various *in vivo* environmental factors, such as nerve growth factor and endogenous EF. In this study, we demonstrated that the morphologic and phenotypic changes of mouse neural stem and progenitor cells (mNPCs) could be induced solely by exposure to square-wave DC pulses (magnitude 300 mV/mm at frequency of 100-Hz). The DC pulse stimulation was conducted for 48 h, and the morphologic changes of mNPCs were monitored continuously. The length of primary processes and the amount of branching significantly increased after stimulation by DC pulses for 48 h. After DC pulse treatment, the mNPCs differentiated into neurons, astrocytes, and oligodendrocytes simultaneously in stem cell maintenance medium. Our results suggest that simple DC pulse treatment could control the fate of NPCs. With further studies, DC pulses may be applied to manipulate NPC differentiation and may be used for the development of therapeutic strategies that employ NPCs to treat nervous system disorders.

## Introduction

Neural stem and progenitor cells (collectively termed *neural precursor cells* or *NPCs*) in the adult mammalian brain are promising candidates for the development of neuroregenerative strategies. NPCs are undifferentiated precursor cells defined by their self-renewal ability, multipotency, and proliferative capacity. Traditionally, NPC differentiation is controlled by the substratum and molecular mediators. For example, cells grown on laminin-coated slides show induced neurite extension and branching [1,2]. *N*-docosahexaenoylethanolamine (synaptamide), an endogenous docosahexaenoic acid metabolite with an endocannabinoid-like structure, promotes neurite growth, synaptogenesis, and synaptic function [3]. However, epidermal growth factor (EGF) and basic fibroblast growth factor (bFGF) are particularly critical for NPC survival and expansion. EGF and bFGF promote NPC proliferation, maintaining the undifferentiated state [4]. Through proliferation and division, NPCs generate clonally related progeny that eventually differentiate to form the neurons, astrocytes, oligodendrocytes, and ependymal cells of the central nervous system (CNS) [5].

Electrical fields (EFs) play important roles in CNS development and tissue repair [6–9]. Various types of electrical stimulation can regulate cell physiologic activities such as division [10], migration [11], differentiation [12], and cell death [13]. EF cathodally directs the turning of growth cones during axon elongation [14,15]. In addition, electrical stimulation has been used in promoting healing in spinal cord repair [16].

Mammalian neurons respond to EFs and supported the notion that neurites are influenced by endogenous EFs during development [17]. Researchers in several studies have reported the effect of exogenous EFs applied to various stem cells *in vitro*. For instance, adult NPCs in a direct current (DC) EF favor neuronal differentiation but have no apparent tendency to differentiate into other neural phenotypes [18]. Biphasic electrical currents also promote the differentiation of fetal neural stem cells (NSCs) into neuronal cells [19]. In these previous studies [18,19], the effect of EF on NPCs was studied by using differentiation medium. In other words, the effects of EF have been observed in the absence of the maintenance factor.

The ability of DC pulses to affect other cell behaviors has been reported. External DC pulses phase-matched to NAD(P)H oscillations promoted neutrophil extension and cytoskeletal shape change [20]. In HT-1080 fibrosarcoma cells, NAD(P)H resonance with pulsed DC EFs led to an increase in the proteolytic activity and reactive oxygen metabolite production. Evidence of damaged DNA has also been observed [21]. Mouse adipose-derived stromal cells drastically reorganize their cytoskeleton, increase the expression of early osteogenic markers, and exhibit enhanced osteogenesis after DC pulse stimulation [22]. As aforementioned, cells are biologically responsive to DC pulses. Therefore, it is important to investigate the effect of DC pulses on NPCs.

The survival, proliferation, and differentiation of NPCs are important for neuroregenerative and functional recovery because NPCs can proliferate and differentiate into neurons. Previous studies have shown the effects of alternating current (AC) EF on the differentiation of NPCs into different types of neurons [23–25]. In these studies, the effects of EF have been observed in the absence of the maintenance factor. Furthermore, NPC differentiation into other neural phenotypes *in vitro* under long-term DC pulse stimulation has never been investigated in stem cell maintenance medium. Here, we evaluated the effects of long-term DC pulses on mouse neural stem and progenitor cell (mNPC) differentiation processes by using stem cell maintenance medium.

## Materials and Methods

### Fabrication of electrotactic chip

**Fig 1:**
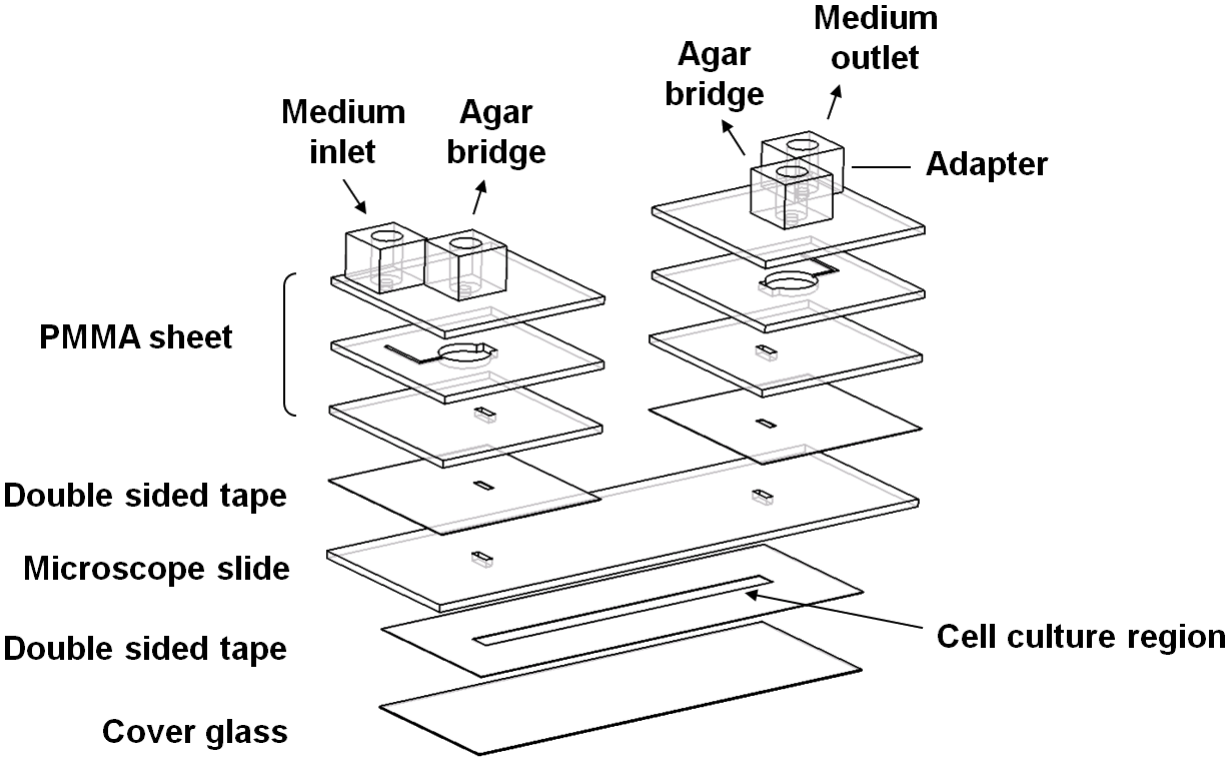
A drawing of the electrotactic chip assembly. The electrotactic chip had connecting holes for the medium inlet and outlet and the agar salt bridges. Cells were cultured in the cell culture region. The width, length, and thickness of the cell culture region were 3 mm, 45 mm, and 70 μm, respectively.

To introduce DC pulses to the mNPCs, a microfluidic chip (the electrotactic chip) was used. Microfluidic chips are very effective in reducing the size of an experiment setup and in turn the reagent and sample consumption. More importantly, the microenvironment of the cells can be precisely controlled. The configuration of the electrotactic chip for differentiation is shown in Fig 1. The detailed fabrication procedure we used is described in our previous studies [26–28]. In brief, the patterns on three polymethyl methacrylate (PMMA) layers were drawn using AutoCAD software (Autodesk, San Rafael, CA) and then cut on a piece of 1-mm-thick PMMA substrate with a CO_2_ laser scriber (ILS-II; LTT Group, Hsin Chu City, Taiwan). The through-holes on the microscope slide were drilled with an ultrasonic driller (Lapidary & Sonic Enterprises, Taipei, Taiwan). The cell culture region was patterned by CO_2_ laser ablation on a piece of 70-μm-thick polyester double-sided tape (PET 8018; 3M, St. Paul, MN). The biocompatibility of the double-sided tape was confirmed in one of our previous studies [29]. The PMMA substrates with the trenches were then bonded with microscope slides (FEA; Yeong-shin, Taipei, Taiwan) using the double-sided tape. After disinfection by ultraviolet light exposure for 30 min, the PMMA chip and the patterned, double-sided tape were adhered to a cover glass (BB024060A1 Deckgläser; Thermo Fisher Scientific Gerhard Menzel, Braunschweig, Germany) to complete the electrotactic chip containing the sealed microchannel.

### Cell culture and preparation

The NPCs were isolated from the cortex of embryonic day 13.5 (E13.5) mice (ICR strain). Groups of female ICR mice aged 8 weeks were purchased from BioLASCO (Taipei, Taiwan). The pregnant mice were sacrificed on the same day. Firstly, pregnant mice were deeply anesthetized with avertin (0.2ml/10g), and then the embryos were harvested at E13.5. Embryos were then transferred to a lab hood where surgery was performed, and the mNPCs (designated as passage 0, P0) were harvested. Each batch of the mNPCs (P1) was labeled with nestin and Sox2 to confirm the expression of the neural stem cell markers. All manipulation and experimental protocols involving animals were approved by the institutional animal care and use committee at Academia Sinica, Taipei, Taiwan.

A complete medium (stem cell maintenance medium) consisting of Gibco Dulbecco’s modified Eagle’s medium/Ham's nutrient mixture F-12 (Life Technologies, Grand Island, NY), 2% Gibco B-27 supplement (Life Technologies), 20 ng/ml EGF (PeproTech, Rocky Hill, NJ), and 20 ng/ml bFGF (PeproTech) was used for the culture of mNPCs in all experiments. Neurospheres were incubated in tissue culture polystyrene flasks (Nunc, Roskilde, Denmark), placed in an incubator filled with 5% CO_2_ atmosphere, and maintained at 37°C. Cells were subcultured every 3–4 days. All experiments were performed with cells that had undergone 3–8 passages from the original source.

**Fig 2:**
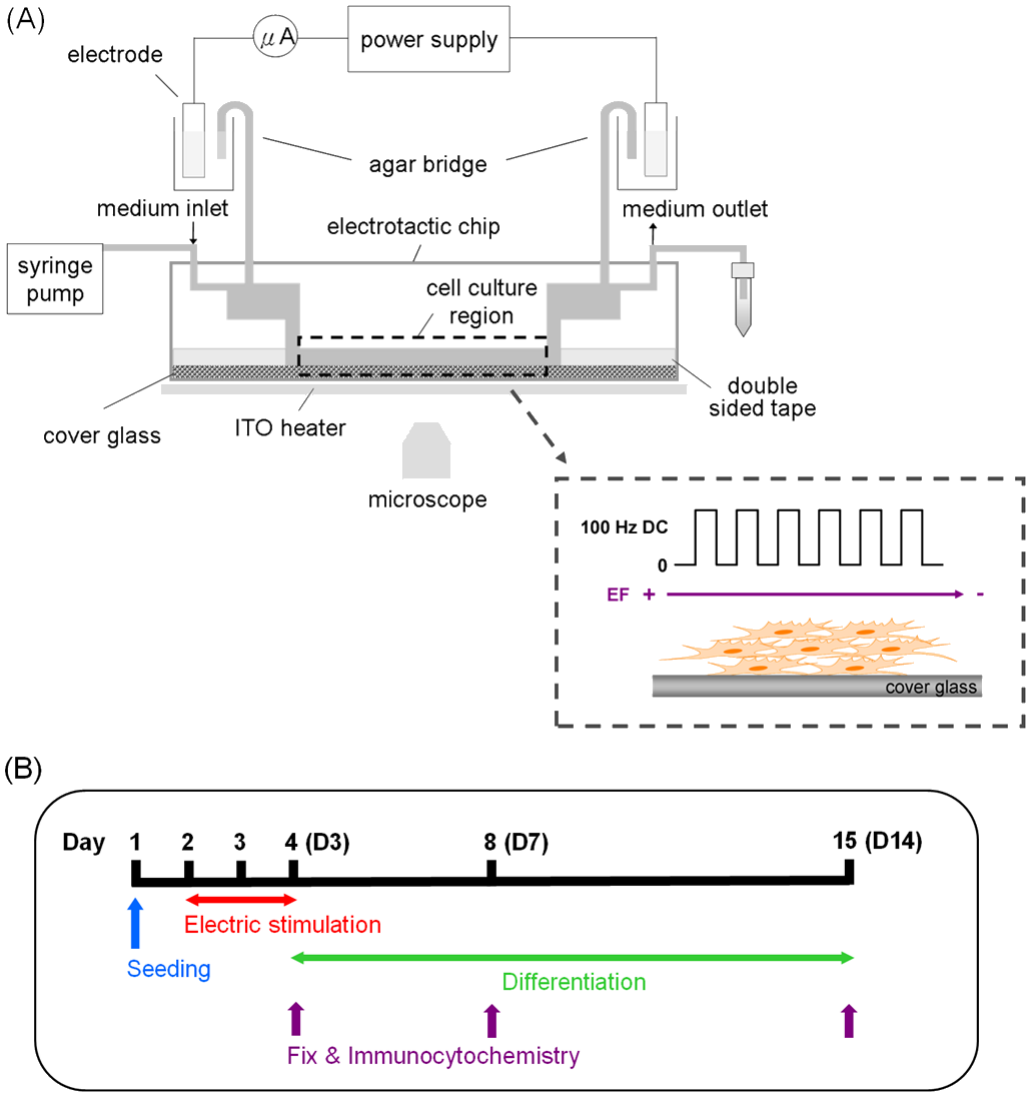
Experimental scheme showing the electrical stimulation during differentiation. (A) The configuration of the entire system for the electrical field stimulation study. (B) Experimental timeline of the electrical stimulation. D3, D7, and D14 denote 3, 7, and 14 d, respectively, of *in vitro* (DIV) culturing after seeding.

The neurospheres (1×10^6^ cells/ml) were suspended in a complete medium containing EGF and bFGF and then infused by manual pumping into the electrotactic chip via the medium outlet. The total volume of the cell suspension was 0.5 ml. The cells were cultured in the electrotactic chip for 4 h for adherence to the poly-l-lysine–coated cover glass (Sigma-Aldrich, St. Louis, MO). Subsequently, fresh complete medium was constantly pumped through the chip via the medium inlet at a flow rate of 20 μl/h using a syringe pump (NE-1000; New Era Systems Inc., Farmingdale, NY), which was connected to the inlet of the electrotactic chip. The cells were then grown and maintained in the chip for an additional 24 h before EF stimulation to allow cell attachment and growth. To monitor the temperature of the microfluidic chip, a K-type thermocouple (TPK-02A; Tecpel, Taipei, Taiwan) was clamped between the chip and the indium–tin–oxide (ITO) heater (Fig 2A; ITO glass, part no. 300739; Merck, Whitehouse Station, NJ). The temperature was controlled with a proportional–integral–derivative (PID) controller (TTM-J4-R-AB; Toho Electronics, Nagoya, Japan) and maintained at 37°C.

### System used for DC pulse stimulation

The DC pulse stimulation system we used consists of the electrotactic chip (Fig 1), the transparent ITO heater, agar salt bridges, a syringe pump, an electrical power supply, a multimeter (Model 189; Fluke Corp., Everett, WA), and an inverted microscope (CKX41; Olympus, Center Valley, PA) equipped with a digital camera (E-330; Olympus) (Fig 2A). For application of the DC pulses to the electrotactic chip, the chip was connected first to the agar salt bridges [1.5% agar dissolved in phosphate-buffered saline (PBS)] and then to the power supply. Ag/AgCl electrodes were used for the electrical connections to the cell culture medium. The choice of the electrode material was based on the fact that the electrolytic product flowing through the cell culture region is chloride ions, which are already abundant in the medium. The power supply was set up as follows. A function generator (33120A; Agilent Technologies, Santa Clara, CA) was connected to an amplifier (A-304; A.A. Lab Systems Ltd. Ramat-Gan, Israel) to output square-wave DC pulses with a magnitude of 300 mV/mm at a frequency of 100-Hz at 50% duty cycle (50% time on and 50% time off). After cell attachment, the mNPCs were subjected to DC pulse stimulation in a continuous manner according to the timeline shown in Fig 2B. For the control condition, the mNPCs were cultured in the chip but did not receive EF stimulation. The cross-sectional area of the cell culture region was 0.07 mm × 3 mm. The electrical current required to generate a continuous EF strength of 300 mV/mm was 86.94 μA. We confirmed that the cell viability did not significantly change. The complete medium was continuously pumped at a rate of 10 μl/h to supply adequate nutrition to the cells and to maintain a constant pH value in the medium. To avoid contamination, the medium was not exposed to air during the experiments. The strength of the applied DC EF has been known to be significant for the differentiation of PC12 cells [30]. Therefore, in our study, the mNPC are subjected to square DC pulses with magnitude of 300 mV/mm at frequency of 100 Hz for 48h.

### Cell viability assay

The cell viability was measured by Molecular Probes SYTOX^®^ Green/Hoechst 33342 staining (Thermo Fisher Scientific, Eugene, OR). SYTOX^®^ Green is a high-affinity nucleic acid stain that easily penetrates cells with compromised plasma membranes but does not cross the membranes of live cells [31]. After 3, 7, or 14 day *in vitro* (DIV) culturing after the seeding, SYTOX^®^ Green (1 μM) and Hoechst 33342 (16.2 μM) was pumped in to the chip at a flow rate of 100 μl/min for 10 min. Cells were then incubated at 37°C for 20 min. After 20 min, the cells are examined using a confocal fluorescence microscope (TCS SP5; Leica Microsystems, Wetzlar, Germany).

### Immunocytochemistry and fluorescence microscopy

To compare undifferentiated and differentiated cells, it is important to evaluate the phenotypic properties of mNPCs under pulsed DC stimulation. The phenotypic markers used in our study included nestin, Sox2, Tuj-1, GFAP, and O4. Nestin is expressed in early neural lineage and differentiated insulin-secreting cells [32,33]. Expression of the transcription factor Sox2 is commonly used as a neural plate or early neural marker and is essential for neurogenesis in the CNS [34]. The immature neuronal Tuj-1 is expressed almost exclusively in early neuronal differentiation [35]. GFAP is expressed in neural tissues and distinguishes astrocytes from other glial cells during CNS development [36]. The oligodendrocyte marker O4 is an antigen on the surface of oligodendrocyte progenitors. O4 has commonly been used as the earliest recognized marker specific for the oligodendroglial lineage [37].

After 3, 7, or 14 DIV culturing after seeding, the cells were rinsed with PBS and fixed with 4% paraformaldehyde (PFA). PBS and PFA were pumped into the chip at a flow rate of 25 μl/min for 20 min. Cells were then permeabilized with 0.1% Triton X-100. Triton X-100 was pumped into the chip at a flow rate of 50 μl/min for 6 min to replace the PBS and PFA. Next, the Triton X-100 was pumped into the chip slowly at a flow rate of 50 μl/h for an additional 30 min to react with the cells. The cells were then blocked with PBS containing 1% bovine serum albumin (BSA) to reduce nonspecific antibody binding. The BSA was pumped at a flow rate of 50 μl/min for 6 min to replace the Triton X-100. The flow rate was next reduced to 100 μl/h and then pumped for 1 h. The cells were subsequently detected by double immunostaining using the following three combinations: (1) nestin (to identify early neural cells) (mouse anti-nestin, 1:1000; abcam, Cambridge, MA) and Sox2 (to identify early neural cells) (rabbit anti-Sox2, 1:1000; abcam), (2) neuron-specific class III beta-tubulin (Tuj1) (to identify neurons) (rabbit anti-Tuj1, 1:500; abcam) and glial fibrillary acidic protein (GFAP) (to identify astrocytes) (mouse anti-GFAP, 1:1000; eBioscience, San Diego, CA), or (3) Tuj1 (to identify neurons) (rabbit anti-Tuj1, 1:500; abcam) and O4 (to identify oligodendrocytes) (mouse anti-O4, 1:500; R&D Systems, Minneapolis, MN). The antibodies for double immunostaining were first pumped into the chip, and then the chip was incubated for 18 h at 4°C. The reagents used for immunostaining were all diluted with PBS. After a washing step with PBS, Alexa Fluor–conjugated secondary antibodies (1:800; Thermo Fisher Scientific) were applied to the cells for 1 h at room temperature (RT) in the dark. Cells were then rinsed with PBS. PBS was pumped at a flow rate of 50 μl/min for 15 min. For nuclear staining, Hoechst 33342 (1:1000) was pumped into the chip and incubated for 10 min at RT. The cells were then washed by pumping PBS into the chip at 50 μl/min for 10 min. After immunostaining, the cells were examined using a confocal fluorescence microscope (TCS SP5).

### Image analysis and data processing

The fluorescent images were analyzed using ImageJ software (National Institutes of Health, Bethesda, MD) with a built-in measurement tool. For cell viability analysis, the integrated fluorescence intensity of SYTOX^®^-stained cells (λ_Ex_ = 488 nm,λ_Em_ = 523 nm) and Hoechst-stained cells (λ_Ex_ = 350 nm, λ_Em_ = 460 nm) were calculated. The cell viability was then determined as cell viability in percent = 100% − (SYTOX^®^ intensity/Hoechst intensity) × 100%. For cell differentiation analysis, the total number of cells (Hoechst-counterstained nuclei) and the number of cells expressing each phenotypic marker were counted. Data were expressed as percentages of cells detected at the same magnification (×40). The total cell number and the percentage of cells expressing each phenotypic marker were compared between the control and treatment groups. All experiments were performed at least three times. Data are expressed as mean ± SEM. Statistical significance was determined by using Student’s *t* test or two-way analysis of variance (ANOVA). *P* < 0.05 represents statistical significance. The asterisk (*) denotes *P* < 0.05, the double asterisks (**) denotes *P* < 0.01, and the triple asterisks (***) denotes *P* < 0.001.

## Results

### Morphologic characteristics of mNPCs in electrical fields

**Fig 3:**
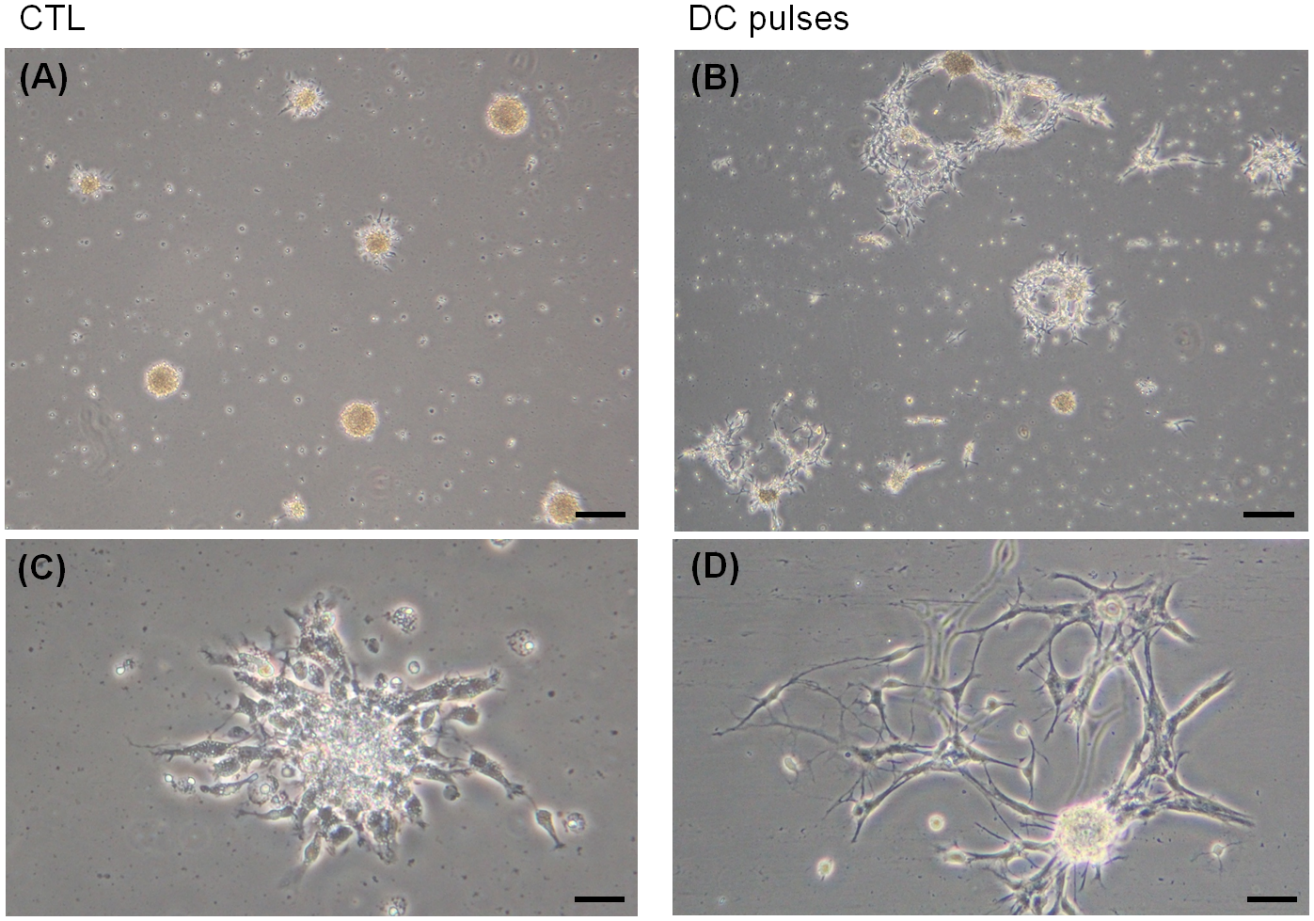
Morphologic characteristics of mouse neural stem and progenitor cells (mNPCs). Morphology of mNPCs without EF stimulation (A and C) and after stimulation (B and D) by DC pulses for 48 h. (A and B) Low-magnification view (×4) and (C and D) high-magnification view (×20). Scale bars: 200 μm (A and B) and 30 μm (C and D).

The cells in the control group (CTL), which were grown in the absence of an electrical field, are shown in Fig 3A and 3C. The undifferentiated mNPCs maintained a round and clustered morphology. However, striking morphologic changes were observed in cells exposed to EF stimulation (Fig 3B and 3D). The primary processes and the branches of mNPCs were clearly evident after stimulation by DC pulses for 48 h. The differentiated polygonal and spread cell morphology was observed. This result showed that cell morphology changed after exposure to DC pulses. In addition, we did not observe the migration of the cells after stimulation by DC pulses for 48 h. To investigate this further, the expression ratios of the phenotypic markers, including nestin, Sox2, Tuj-1, GFAP, and O4, were analyzed.

### Effect of electrical fields on mNPC viability and proliferation

**Fig 4:**
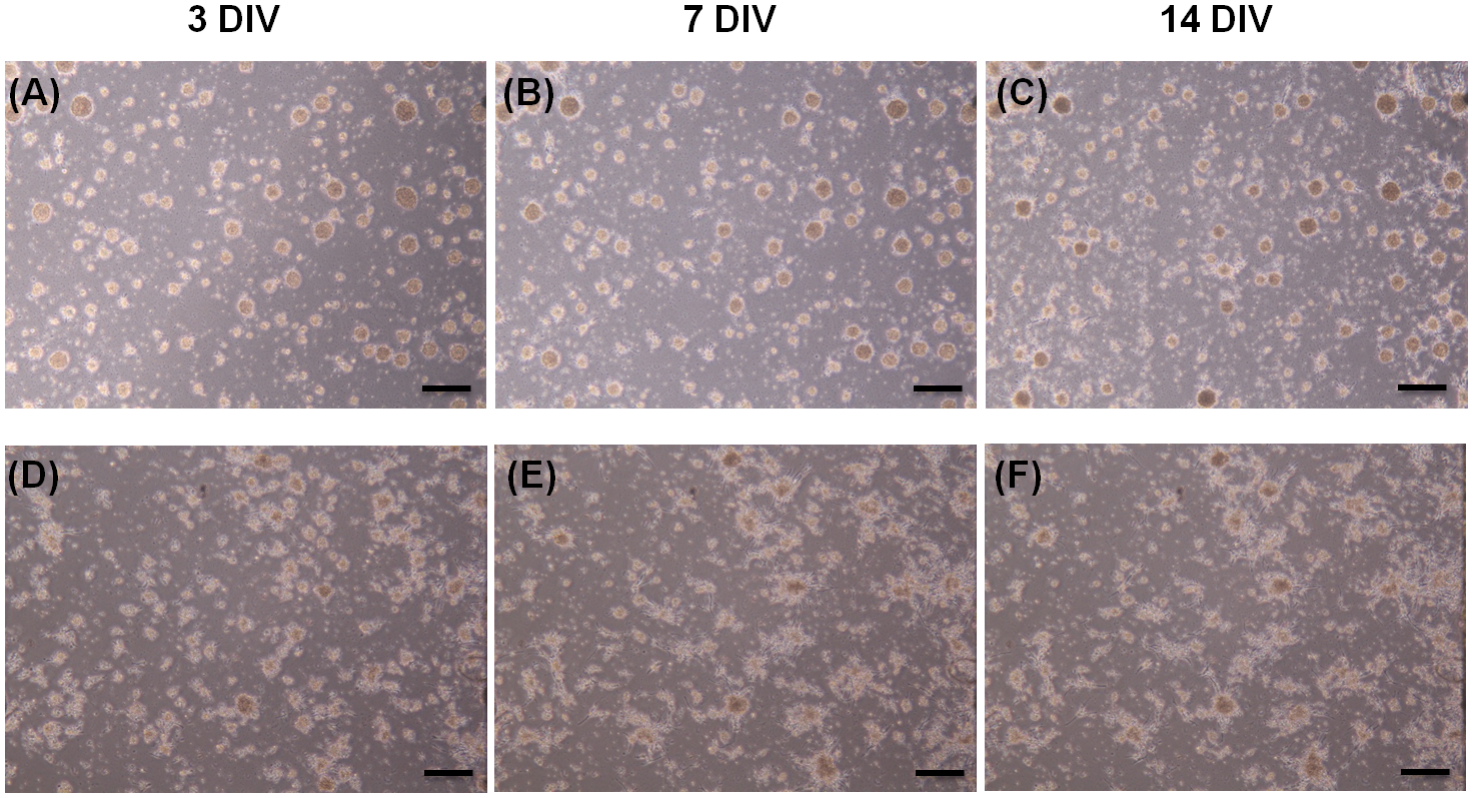
Cell morphology in the control and electrical field (EF) groups after 3, 7, and 14 d *in vitro* (DIV). Morphology of mNPCs without EF stimulation (A–C) and after stimulation (D–F) by the DC pulses. The images were taken at 3 DIV (A and D), 7 DIV (B and E), and 14 DIV (C and F). Low-magnification view (×4). Scale bars: 120 μm.

**Fig 5:**
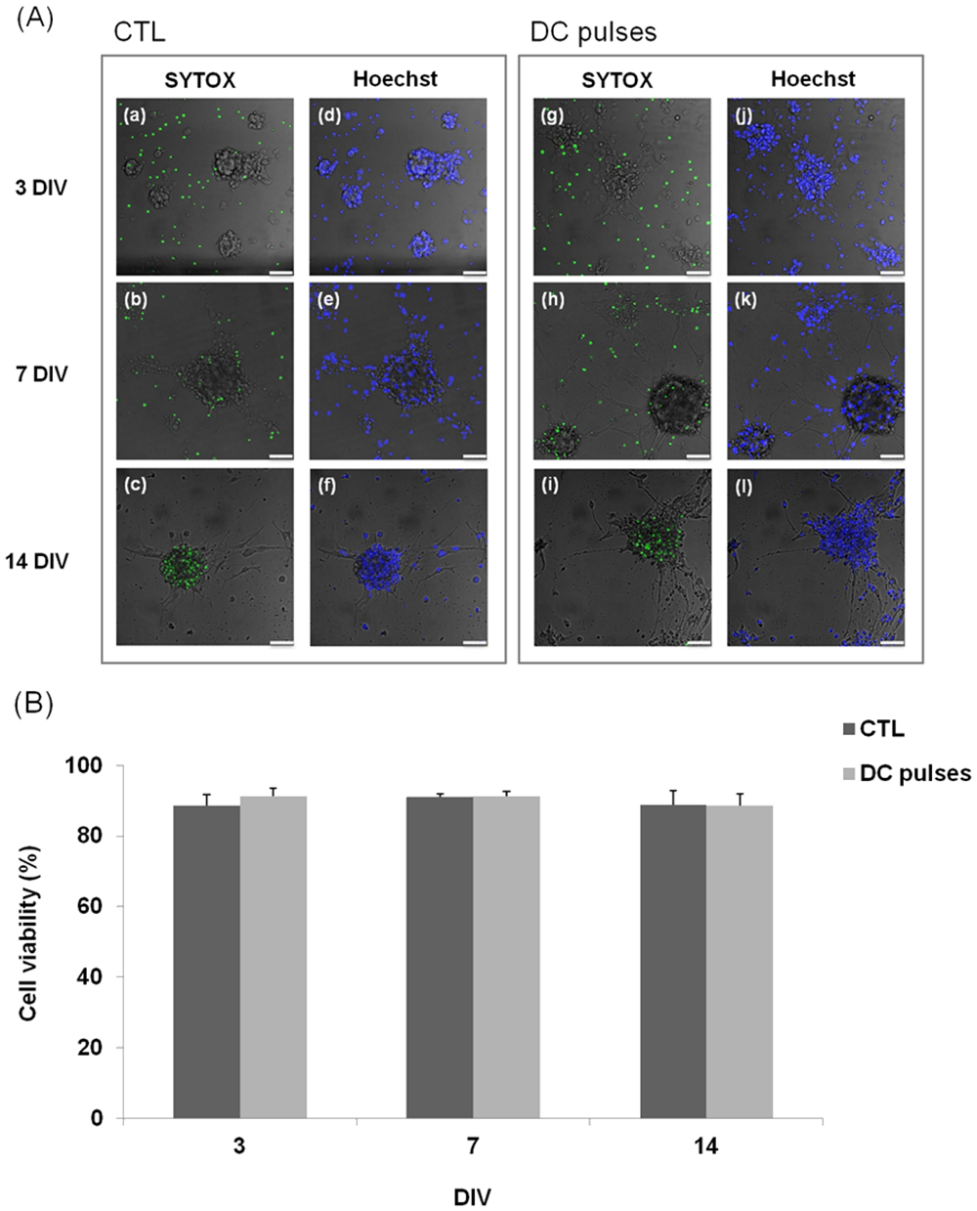
Effect of electrical fields (EFs) on mouse neural stem and progenitor cell (mNPC) viability. (A) The cells were labeled with SYTOX^®^ (green) and Hoechst 33342 (blue). (a–f) No EF stimulation. (g–l) With DC pulses. Scale bars: 50 μm. (B) Cell viability of the control and EF groups after 3, 7, and 14 d *in vitro*.

The neurospheres were grown in the electrotactic chip and cultured without (CTL) or with (EF) square-wave DC pulse stimulation. The cell morphologies of the CTL and EF groups after 3, 7, and 14 DIV are shown in Fig 4. The cells had a neurite-like structure that surrounded the sphere and showed good attachment to the substrate surface. The cell viability was good in both the CTL and EF groups. There was no significant change in cell viability of mNPCs after 3, 7, and 14 DIV in both groups (Fig 5).

### Electrical fields enhanced expression of nestin

**Fig 6:**
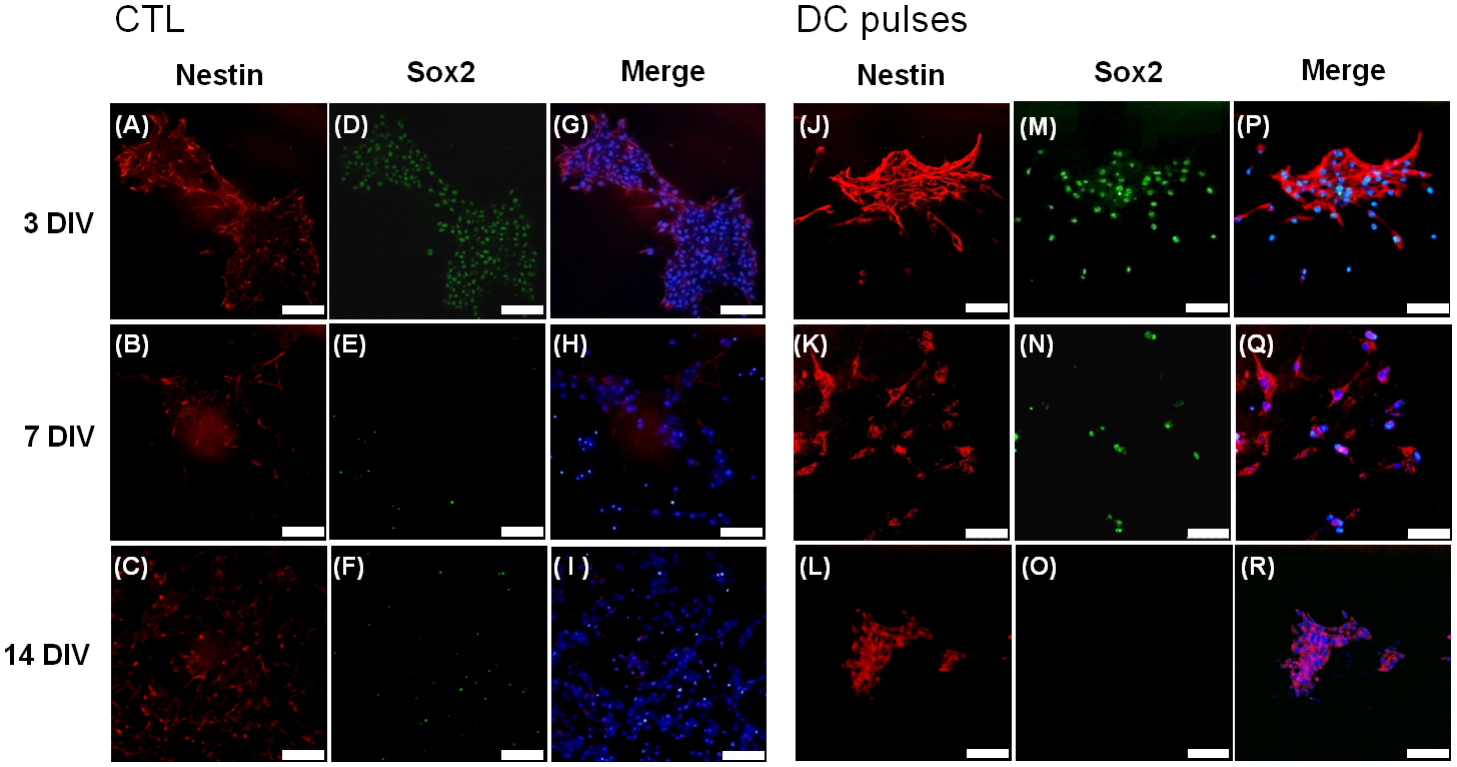
Immunofluorescence characterization of mouse neural stem and progenitor cells (mNPCs). The cells were labeled with nestin (red), Sox2 (green), and Hoechst 33342 (blue). (A–I) Without EF stimulation. (J–R) With square-wave DC pulses. Scale bars: 75 μm.

**Fig 7:**
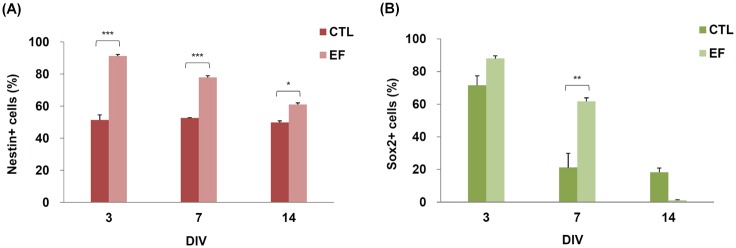
The expression of nestin and Sox2 without and with DC pulse stimulation. The percentage of immunostained cells with (A) nestin and (B) Sox2 for mouse neural stem and progenitor cells (mNPCs) cultured with (electrical field [EF]) and without (control [CTL]) square-wave DC pulse stimulation. The *P* values of Sox2 expression at 3 and 14 DIV for the CTL and EF groups were 0.080 and 0.071, respectively.

Fluorescent images of the phenotypic markers are shown in Fig 6. The CTL cells were grown in the stem cell maintenance medium and in the absence of EF. In the CTL group, over 50% of the cells expressed nestin and Sox2 (nestin+, 51.3 ± 3.2%; Sox2+, 71.6 ± 5.8%) at 3 DIV, whereas Sox2 expression was reduced (Sox2+, 18.3 ± 2.6%) at 14 DIV. Expression of nestin was extremely high at all time points (3 DIV, 91.2 ± 0.0%; 7 DIV, 77.9 ± 2.9%; 14 DIV, 61.0 ± 1.1%) in the stimulation groups. In addition, the expression of Sox2 was observed in the stimulation group at 3 DIV. The results shown in Fig 6 are quantitatively presented in Fig 7. These results show that the DC pulses enhanced the expression of nestin at an early stage (3 DIV).

### Electrical field–induced expression of differentiation markers

**Fig 8:**
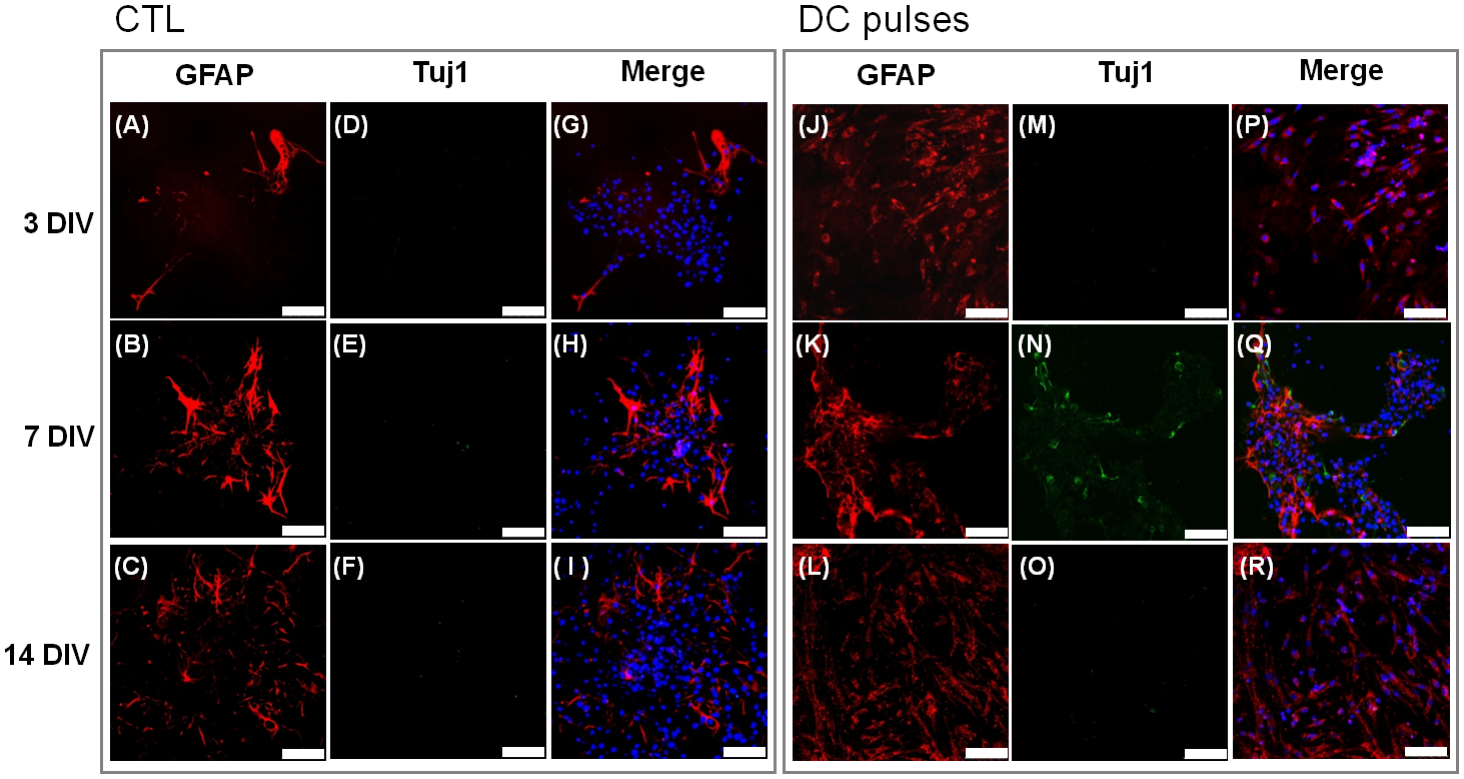
Fluorescence microscopy images of immunostained mouse neural stem and progenitor cells (mNPCs) labeled with glial fibrillary acidic protein (red), Tuj1 (green), and Hoechst 33342 (blue). (A–I) mNPCs without electrical field (EF) stimulation. (J–R) mNPCs subjected to square-wave DC pulses. Scale bars: 75 μm.

**Fig 9:**
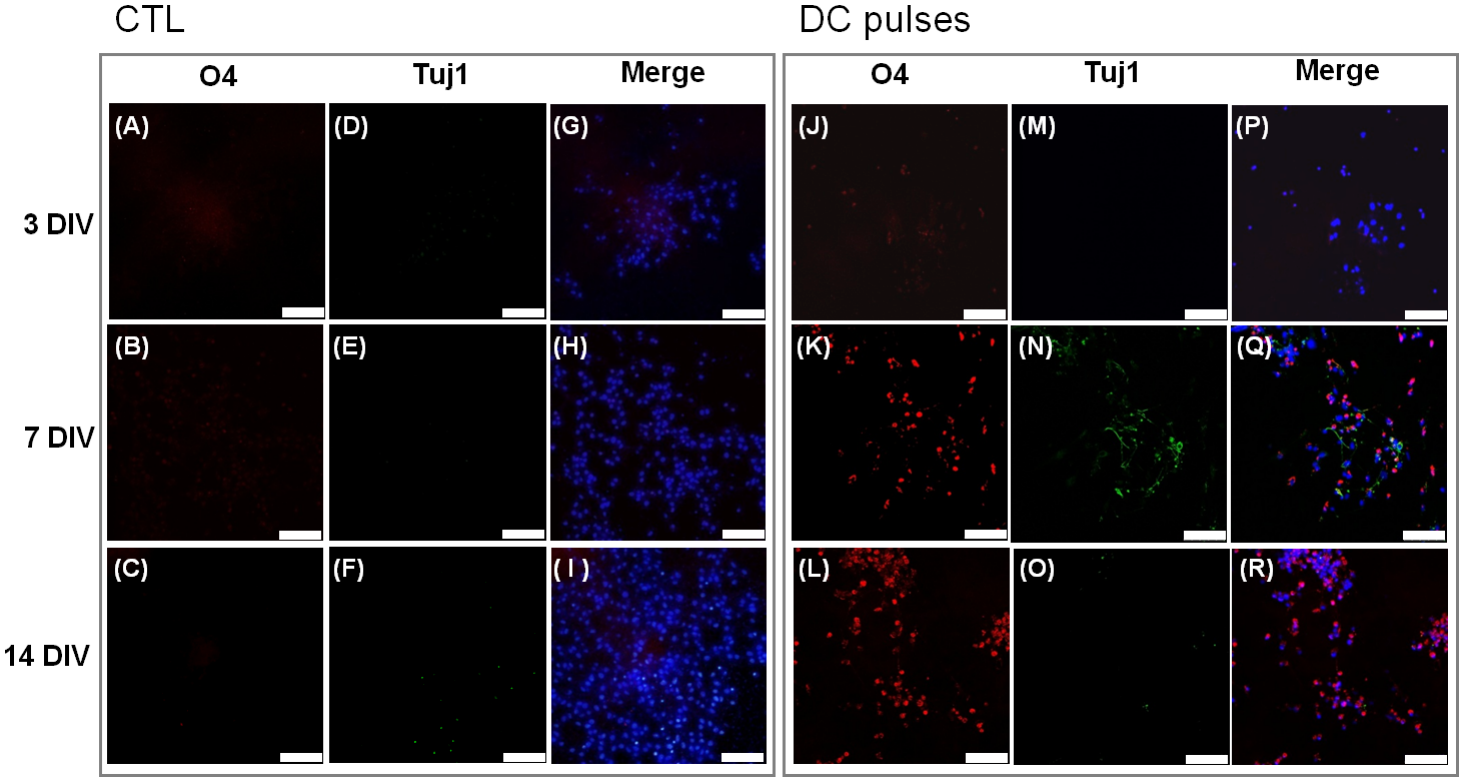
Fluorescence microscopy images of immunostained cells labeled with O4 (red), Tuj-1 (green), and Hoechst 33342 (blue). (A–I) mouse neural stem and progenitor cells (mNPCs) without electrical field stimulation. (J–R) mNPCs subjected to square-wave DC pulses. Scale bars: 75 μm.

**Fig 10:**
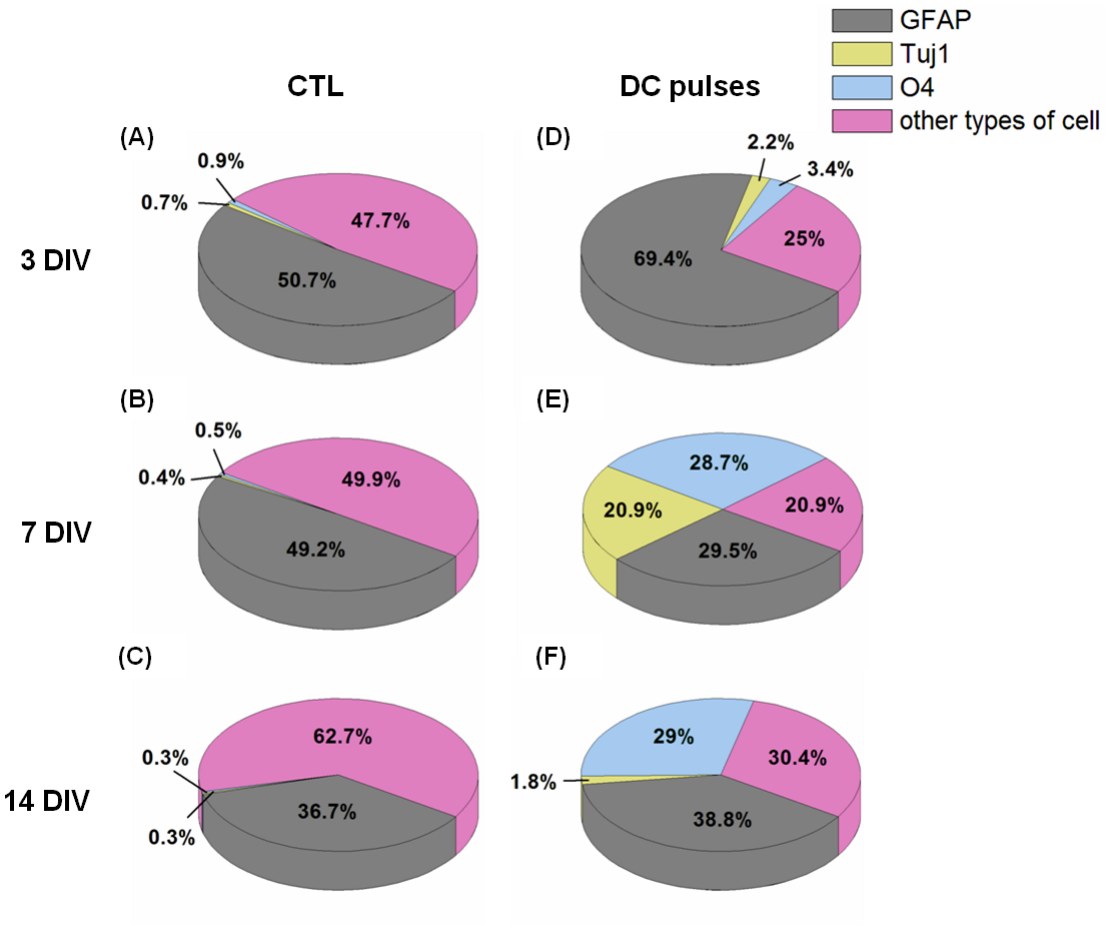
Percentage of immunostained cells with Tuj1 (neurons), glial fibrillary acidic protein (astrocytes), and O4 (oligodendrocytes). Differentiation of mouse neural stem and progenitor cells (A–C) in control conditions and (D–F) with electrical field stimulation after 3 (A and D), 7 (B and E), and 14 (C and F) d *in vitro*, respectively.

The differentiation fate of the cells was observed on fluorescent micrographs, which showed immunostained cells for Tuj1, GFAP, and O4 (Figs 8–10). GFAP+ cells (astrocytes) were present in the CTL group (Fig 8A and 8C) and in the stimulation groups (Fig 8J–8L) at 3, 7, and 14 DIV. In the stimulation group, the number of Tuj1+ cells (neurons) increased to a high ratio at 7 DIV (Fig 8N) but decreased at 14 DIV (Fig 8O).

O4+ cells (oligodendrocytes) were present at high ratios in the stimulation group at 7 and 14 DIV (Fig 9K and 9L) but at low ratios in the CTL groups at 3, 7, and 14 DIV (Fig 9A–9C). Consistent with the results from the GFAP-Tuj1 staining (Fig 8N), Tuj1+ cells (neurons) were present at high ratios in the stimulation group at 7 DIV (Fig 9N).

The results shown in Figs 8 and 9 are quantitatively presented in Fig 10. After the electrical stimulation, mNPCs expressed relatively high levels of GFAP compared with the CTL group at 3 DIV (Fig 10D). Neurons were present at significantly high ratios (Tuj1+, 20.9 ± 4.9%) in the stimulation group at 7 DIV (Fig 10E). At 7 and 14 DIV, O4+ cell numbers were significantly higher (28.7 ± 9.7% and 29.0 ± 5.5%, respectively) in the stimulation groups (Fig 10E and 10F) than in the CTL group. Our results show that long-term continuous DC pulses enhanced the expression of nestin up to 3 DIV and promoted the expression of Tuj1 and O4 simultaneously at 7 DIV.

## Discussion

### DC pulses promoted elongated processes of mNPCs

Several studies have shown that EFs induce neurite elongation of neural cells [2,38–40]. In the work by Hinkle *et al*., *Xenopus* spinal neurons were cultured in the presence of EFs. *Xenopus* neural cells sprouted neurites when exposed to an EF [38]. In the work by Patel *et al*., the number of neurites and the average neurite length were increased by EF stimulation on cultured *Xenopus* embryonic neurons [39]. In the work by Li *et al*., steady and pulsed DC EF stimulation directed the neurite extension of spiral ganglion neurons [2]. In the work by Lee *et al*., some neurites extended after AC/DC stimulation on human NSCs cultured in differentiation medium [40]. In our study, the primary processes and the branches of mNPCs were clearly evident after stimulation by DC pulses for 48 h. Fig 3 demonstrates our finding that DC pulses promoted the elongated processes of mNPCs. The effect of DC pulses is similar but not identical (see below) to that induced by DC stimulation in this regard. To further investigate the effect of the DC pulse stimulation, we first examined the viability of the mNPCs.

### DC pulses did not have an observable effect on mNPC survival and proliferation

In the work by Ariza *et al*., one might anticipate a decrease in proliferation of NPCs in a stronger (437 mV/mm) EF applied for a similar duration. It seems that the accumulation of ions or electrode by-products in the media may begin to negatively affect the cells [18]. In our study, the complete medium was continuously pumped at a rate of 10 μl/h to supply adequate nutrition to the cells and to maintain a constant pH value in the medium. Therefore, cell viability did not change under DC pulse stimulation. As shown in Fig 4, there is no apparent change in cell viability of the mNPCs. Panels (c) and (i) of Fig 5A show that the scattered single cells diminished after 7 DIV and that some dead cells were observed in the sphere. However, cell viability did not change under DC pulse stimulation. As shown in Fig 5B, both the CTL and EF groups showed viability of approximately 88%, indicating good cell growth inside the electrotactic chip. In summary, DC pulse stimulation did not have an observable effect on survival and proliferation of the mNPCs. On the basis of this finding, the effect of DC pulse stimulation on cell differentiation was investigated as described below.

### DC pulses induced differentiation of mNPCs

In our study, immunostaining verified that expression of nestin was enhanced after DC pulse stimulation. Specifically, this stimulation enhanced the expression of nestin until 14 DIV. In contrast to the study by Lim *et al*. [25], in which pNPCs were exposed to daily (4 h/d) stimulation of EF (1 V/cm) at 1 Hz, 10 Hz, and 50 Hz, we found nestin expression in all groups at 3 and 7 DIV but not at 14 DIV. Apparently, compared with the pNPCs in the previous study, the mNPCs in our study showed higher nestin expression at 14 DIV. In addition, Sox2 (88.0 ± 1.6%) (Fig 7) and GFAP (69.4 ± 4.0%) (Fig 10) also demonstrated prominent expression at 3 DIV. In the previous studies [32,33], nestin was expressed in early neural lineages and differentiated insulin-secreting cells. Previous studies have shown that, in the mouse nervous system, Sox2 is expressed in stem cells and early precursors as well as in a few mature neurons [41,42]. In the work by Cavallaro *et al*., Sox2 was important at early stages of NSC differentiation *in vitro*. Sox2 might participate in different networks of transcription factors in stem versus differentiated cells [34]. Because nestin and Sox2 expression is usually absent in differentiated cells [43], the expression of nestin and Sox2 with 100-Hz stimulation suggests that a relatively large portion of cells are still in the process of differentiation [32,34].

### Effects of DC pulses on mNPC differentiation processes in stem cell maintenance medium

The nervous system is composed of neurons and neuroglia. Glial cells compose a network of tissue that provides nutrition and operational support for neurons [44]. Previous studies have shown the effects of EFs on the differentiation of NPCs into different types of cells in differentiation medium that excludes EGF and bFGF [18,19,25]. In the work by Ariza *et al*., NPCs were stimulated with DC EF (437 mV/mm) for 16–24 h/d for the first 3 d and on the final day of each experiment. The cells differentiated into neurons but not into astrocytes or oligodendrocytes [18]. In the work by Chang *et al*., fetal NSCs were exposed to 100-Hz electrical stimulation at a magnitude of 4 μA/cm^2^ with 200-μs pulses. The ratios of neuronal cells were increased by biphasic electrical currents (BECs), but the ratio of astrocytes was slightly decreased. This previous study showed that BEC promotes differentiation of fetal NSCs into neuronal cells, but no results regarding oligodendrocytes were reported [19]. In the work by Lim *et al*., the ratios of neurons and astrocytes were altered after EF stimulation, but no oligodendrocytes were observed. Their study also showed that EF stimulation of higher frequency appears to delay differentiation into mature astrocytes [25].

The purpose of our study was to evaluate the effects of long-term DC pulse stimulation on mNPC differentiation processes. Our results showed that the electrical stimulation enhanced Tuj1, GFAP, and O4 expression in mNPCs, indicating the differentiation into three corresponding types of cells: neurons, astrocytes, and oligodendrocytes. More than 69% of astrocytes were observed at 3 DIV. Significant ratio of neuron cells were observed at 7 DIV. However, the ratios of oligodendrocytes were significantly higher than those in the CTL group at 7 and 14 DIV. In agreement with previous studies [19,25], the ratios of neuronal cells were increased after DC pulse stimulation. We also observed an increase in the ratio of astrocytes and oligodendrocytes. Within the CNS, oligodendrocytes form a myelin sheath of axons that supports rapid nerve conduction [45]. Previous studies have shown that EGF and bFGF promote cell proliferation and inhibit the differentiation of spinal cord oligodendrocyte precursors [4,46]. However, in our study, a significant amount of oligodendrocytes (29% at 14 DIV) were induced after DC pulse stimulation. These findings confirmed that the treatment of simple DC pulses promoted cell differentiation into neurons, astrocytes, and oligodendrocytes simultaneously, even in stem cell maintenance medium containing EGF and bFGF. Obviously, in the presence of the maintenance factor, the DC pulse stimulation resulted in the differentiation into more diversified cell types than by using other types EF stimulations in the absence of the maintenance factor.

Compared with chemically (such as vitamin A) induced differentiation, EF stimulation has the advantage of better control of the stimulation region. For example, using needle electrodes would allow the introduction of EF stimulation to a localized group of cells. In other words, this type of stimulation would allow better spatial control over the stimulation. Another expected advantage is the better temporal control of the stimulation when using EF. EF stimulation can be applied or stopped immediately after turning the external power supply on or off. Stimulation to the cells could thus be manipulated in a time-controlled manner. Such an advantage could not easily be achieved by using chemicals.

Previous studies have reported that PI3K, Akt, RhoA, Cdc42, and Rac1 play an important role in neuronal differentiation [47,48]. It is also interesting to notice that many of the above-mentioned proteins, e.g. Akt, RhoA and Rac1, have been known to participate in the EF-induced cell migration [27,49–51]. In the future, we will study the effects of DC pulses on the expression of the above-mentioned proteins.

To summarize, mNPCs were exposed to square-wave DC pulses with a magnitude of 300 mV/mm at a frequency of 100-Hz. EF stimulation enhanced the expression of nestin up to 3 DIV. In addition, the EF stimulation induced mNPC differentiation to produce astrocytes, neurons, and oligodendrocytes at 3 and 7 DIV, respectively. This result might prove valuable for future clinical application.

## Conclusions

In this study, we investigated the morphologic and phenotypic changes of mNPCs exposed to square-wave DC pulses with a magnitude of 300 mV/mm at a frequency of 100-Hz. Our novelty is twofold: (1) we used DC pulse stimulation and (2) the NPC stimulated was carried out in a stem cell maintenance medium. We have shown that long-term continuous DC pulses enhanced the expression of nestin up to 3 DIV. In addition, the mNPCs expressed Tuj1, GFAP, and O4 after DC pulse stimulation. Our results show that the treatment of simple DC pulses induced mNPC differentiation into neurons, astrocytes, and oligodendrocytes simultaneously in a stem cell maintenance medium that contained EGF and bFGF. The DC pulse stimulation resulted in a more diversified cell types than by using other types EF stimulations in the absence of the maintenance factor.

With further studies designed to better understand the mechanism of DC pulses -induced differentiation, we may be able to manipulate NPC differentiation. The knowledge gained could be used for the development of therapeutic strategies that employ NPCs to treat CNS disorders.
